# Supplementary material for: Discovery of long-range inhibitory signaling to ensure single axon formation
Source: Nat Commun. 2017 Jun 26;8:33. doi: 10.1038/s41467-017-00044-2 (PMC5484694; doi:10.1038/s41467-017-00044-2)
Supplement: Supplementary file 1 — Supplementary Information [file 41467_2017_44_MOESM1_ESM.pdf]

File Name: Supplementary Information
Description: Supplementary Figures and Supplementary Tables

File Name: Supplementary Movie 1
Description: **Local application of NT-3 generates long-range Ca^{2+} waves**. A Ca^{2+} indicator (Cal-520) was loaded into hippocampal neurons at 3 DIV. The pseudocolored image represents the concentration of Ca^{2+} after local application of PBS to the axon terminal. Frames were obtained every 2 sec.

File Name: Supplementary Movie 2
Description: **Local application of NT-3 generates long-range Ca^{2+} waves**. A Ca^{2+} indicator (Cal-520) was loaded into hippocampal neurons at 3 DIV. The pseudocolored image represents the concentration of Ca^{2+} after local application of NT-3 to the axon terminal. Frames were obtained every 2 sec.

File Name: Supplementary Movie 3
Description: **Spatio-temporal dynamics of RhoA activation in hippocampal neurons**. Raichu-RhoA-CR was transfected into hippocampal neurons at 3 to 4 DIV. The pseudocolored image represents the FRET efficiency after local application of PBS to the axon terminal. Frames were obtained every 20 sec.

File Name: Supplementary Movie 4
Description: **Spatio-temporal activation of RhoA in hippocampal neurons**. Raichu-RhoA-CR was transfected into hippocampal neurons at 3 to 4 DIV. The pseudocolored image represents the FRET efficiency after local application of NT-3 to the axon terminal. Frames were obtained every 20 sec.

File Name: Supplementary Movie 5
Description: **Photoactivation of Rho-kinase induces membrane blebbing and cell contraction**. mCherry-Rho-kinase-CAT-Zdk1 was co-transfected with NTOM20-LOV2 into HeLa cells. Photoactivation of LOVTRAP Rho-kinase was accomplished by irradiating intermittently (5 sec on/off cycle) for 30 minutes.

File Name: Supplementary Movie 6
Description: **Photoactivation of LOVTRAP RhoA/Rho-kinase induces minor neurite retraction**. mCherry-Zdk1 (LOVTRAP-Cont) was cotransfected with NTOM20-LOV2 into hippocampal neurons at 3 to 4 DIV. The cell body within a 20-μm square was irradiated every 5 sec. Frames were also obtained every 5 sec.

File Name: Supplementary Movie 7
Description: **Photoactivation of LOVTRAP RhoA/Rho-kinase induces minor neurite retraction**. A kinase dead (KD) mutant of LOVTRAP Rho-kinase was transfected into hippocampal neurons at 3 to 4 DIV. The cell body within a 20-μm square was irradiated every 5 sec. Frames were also obtained every 5 sec.

File Name: Supplementary Movie 8
Description: **Photoactivation of LOVTRAP RhoA/Rho-kinase induces minor neurite retraction**. LOVTRAP-Rho-kinase was transfected into hippocampal neurons at 3 to 4 DIV. The cell body within a 20-μm square was irradiated every 5 sec. Frames were also obtained every 5 sec.

File Name: Supplementary Movie 9
Description: **Local application of Rho-kinase inhibitor to the minor neurite induces neurite outgrowth**. DMSO was locally applied to a minor neurite of a polarized neuron. Frames were also obtained every 5 min.

File Name: Supplementary Movie 10
Description: **Local application of Rho-kinase inhibitor to the minor neurite induces neurite outgrowth**. Rho-kinase inhibitor (Y-27632) was locally applied to a minor neurite of a polarized neuron. Frames were also obtained every 5 min.

File Name: Peer Review File
Description:

Supplementary information

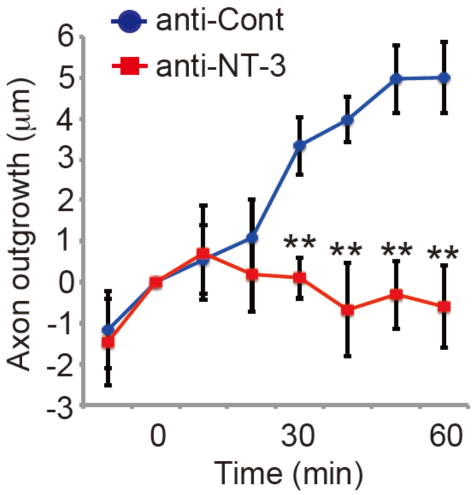

Supplementary Figure 1. NT-3 derived from cultured neurons is required for axon formation in stage 3 cultured hippocampal neurons Local inhibition of NT-3 suppressed axonal elongation. Time course of changes in the lengths of the axon from a single neuron locally exposing with anti-Cont (blue) or anti-NT-3 (red) (anti-Cont= 13, anti-NT-3= 13 neurons from 3 independent experiments). Error bars represent SEM. **P < 0.01.

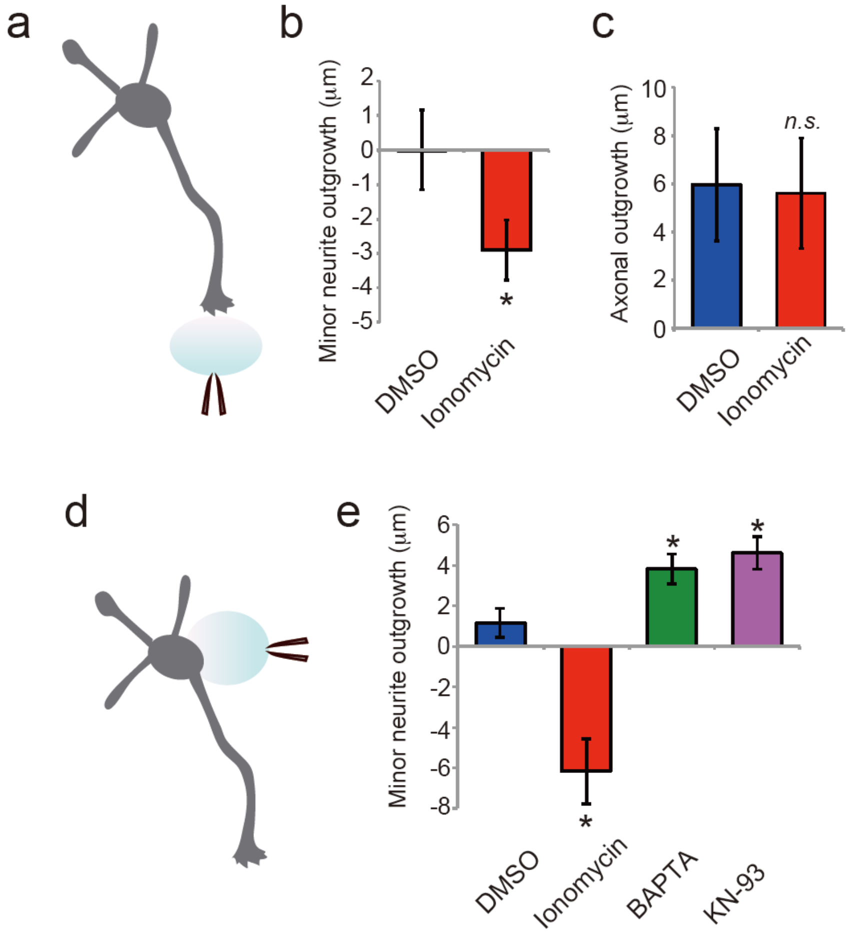

Supplementary Figure 2. Local elevation of Ca^{2+} signaling at the cell body regulates neuronal polarity (**a**) Local application of a Ca^{2+} ionophore to an axon induced minor neurite retraction. DMSO or a Ca^{2+} ionophore (ionomycin) was locally applied to the axon of a polarized hippocampal neuron. (**b-c**) Minor neurite outgrowth and axonal outgrowth were measured (DMSO= 67, ionomycin= 30 neurites from 3 independent experiments). (**d-e**) Local application of a Ca^{2+} ionophore or Ca^{2+} signaling inhibitors to the cell body regulated minor neurite outgrowth. DMSO, ionomycin, BAPTA or KN-93 was locally applied to the cell body of a polarized hippocampal neuron. Minor neurite outgrowth was measured (DMSO=59, ionomycin=33, BAPTA= 24, KN-93= 21 neurites from 3 independent experiments). Error bars represent SEM. *$P < 0.05$ and **$P < 0.01$.

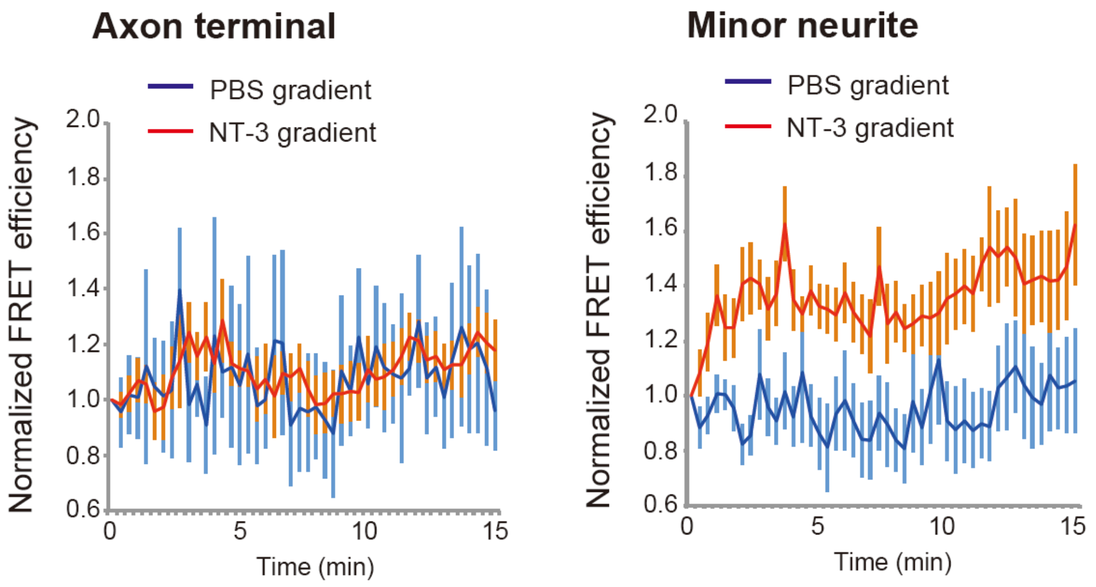

Supplementary Figure 3. Spatio-temporal activation of RhoA by local application of NT-3 in neurons
PBS (blue) or NT-3 (red) was locally applied to an axon terminal of a hippocampal neuron expressing Raichu-RhoA-CR. Time course of changes in FRET efficiency in the axon terminal (left) and the minor neurite (right). Error bars represent the SEM.

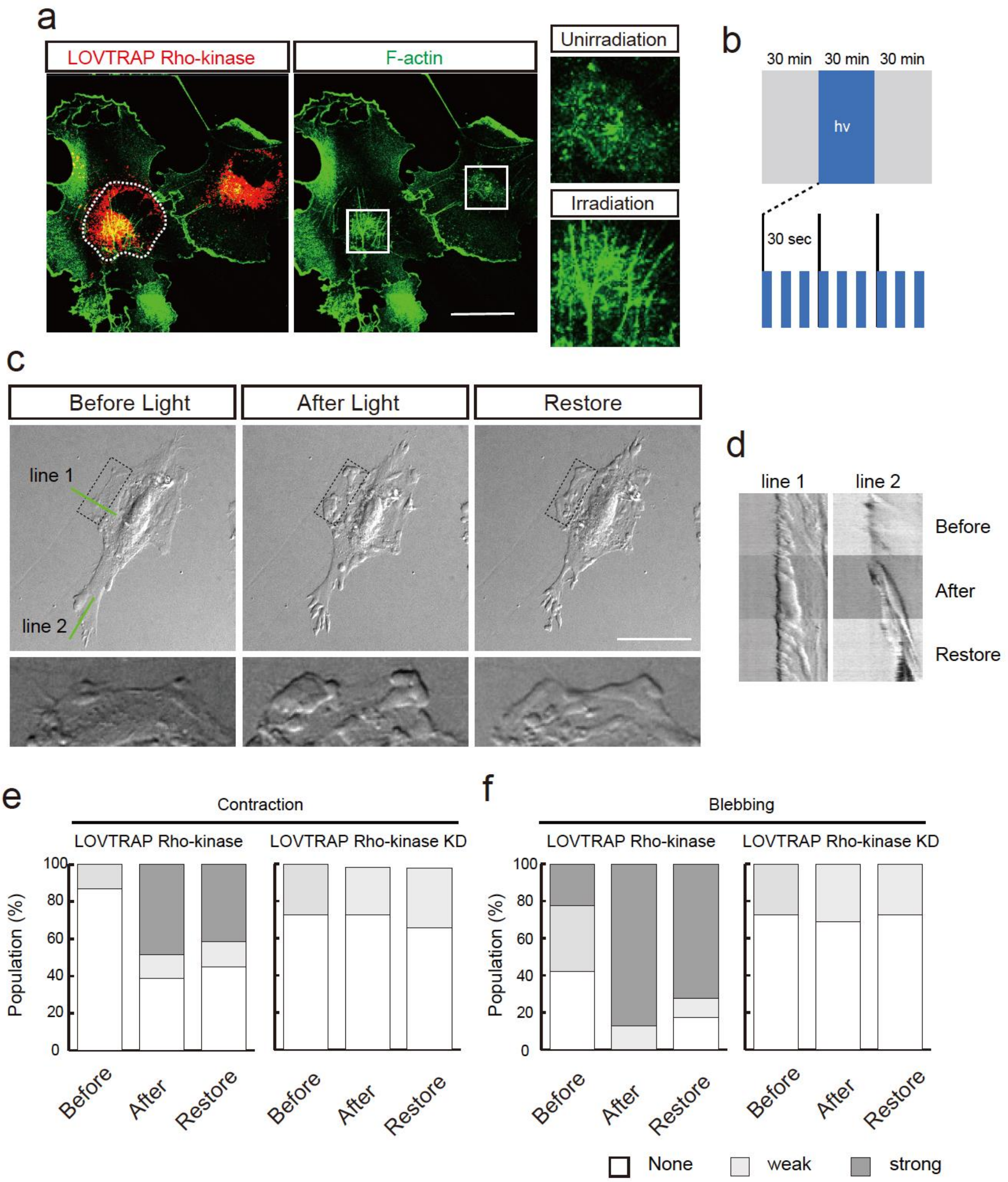

Supplementary Figure 4. Characterization of LOVTRAP-Rho-kinase (**a**) LOVTRAP-Rho-kinase induced stress-fiber formation. COS-7 cells expressing LOVTRAP-Rho-kinase were illuminated at 488 nm in the perinuclear region (dotted lines) for 30 min and then immunostained with Alexa-Phalloidin (green). Scale bars, 50 µm. (**b**) Experimental design. HeLa cells expressing LOVTRAP-Rho-kinase were imaged for 30 min prior to photoactivation. Photoactivation of LOVTRAP-Rho-kinase was accomplished by irradiating intermittently (5 sec on/off cycle) for 30 min. Following photoactivation, the cells were imaged for an additional 30 min. hv, irradiation. (**c**) Irradiation of HeLa cells expressing LOVTRAP-Rho-kinase. High-magnification images of the area indicated by the dotted lines are presented. Scale bars, 40 µm. (**d**) Kymographs of the membrane (line 1 or line 2) during observation. (**e-f**) Percentage of cells exhibiting contraction (e) or membrane blebbing (f).

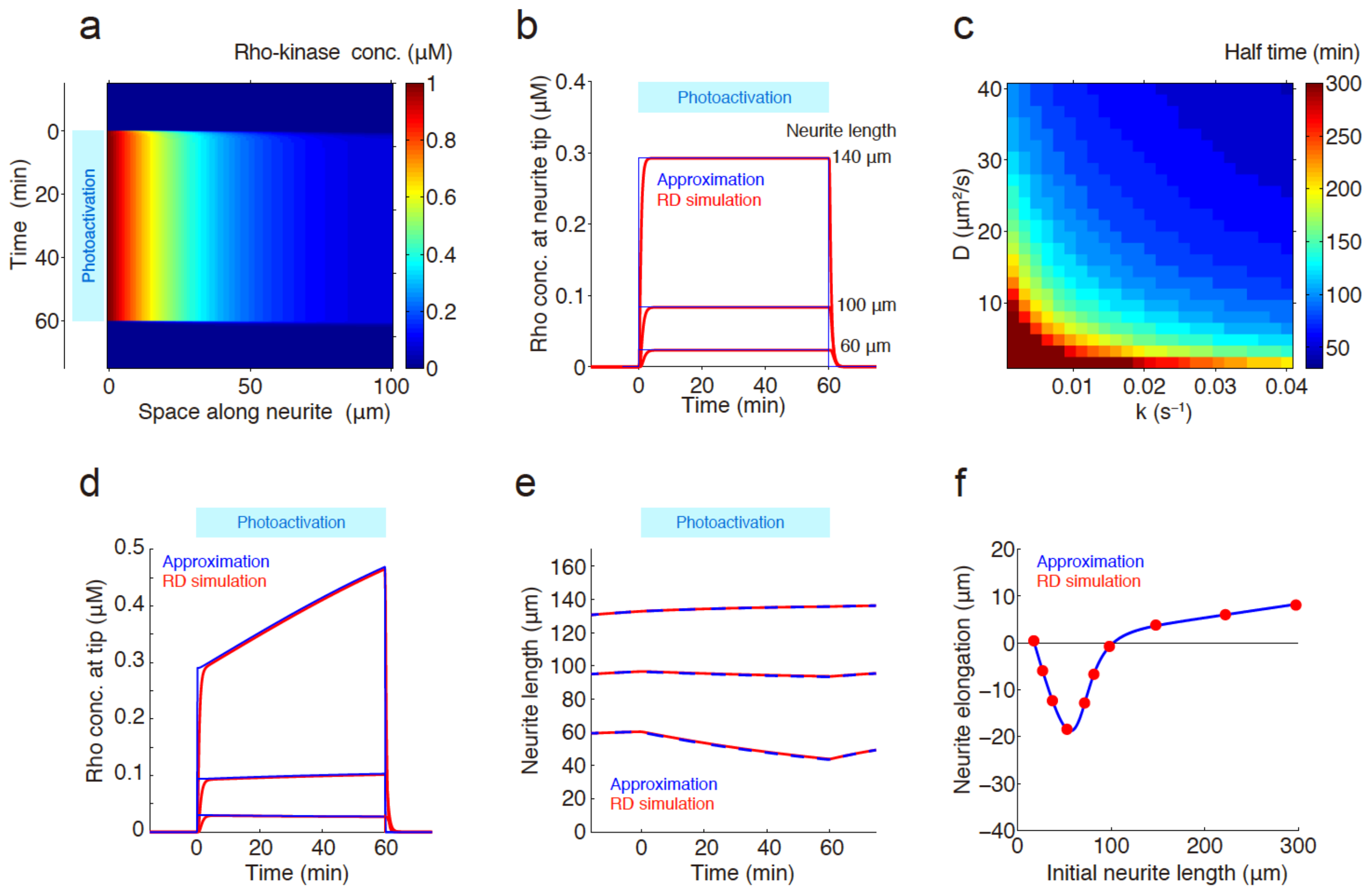

Supplementary Figure 5. Reaction-diffusion (RD) simulation without the steady state approximation (**a, b, c**) Reaction-diffusion process of the Rho-kinase was simulated, without the steady state approximation, along a neurite of fixed length (100 μm). (**a**) The heat map represents a spatiotemporal profile of the Rho-kinase concentration in response to the photoactivation. (**b**) RD simulation as in (a) and approximated simulation as in Fig. 4 were compared with various lengths of neurites. Red and blue lines indicate temporal changes in the Rho-kinase concentration at a neurite tip, which were calculated by the RD simulation as in (a) and by equation (1), respectively. (**c**) The time required for Rho-kinase concentration at a neurite tip (100 μm) to reach half the stationary value from the onset of photoactivation was evaluated upon varying the inactivation/degradation rate, k, and the diffusion rate, D. (**d, e, f**) RD process of the Rho-kinase was simulated along a moving neurite. (**d**) Red and blue lines indicate the Rho-kinase concentration at a neurite tip simulated by the RD simulation and the approximated simulation, respectively. (**e**) Red and blue lines indicate the neurite lengths simulated by RD simulation and the approximated simulation, respectively. (**f**) Blue line indicates the relationship between initial neurite length and LOVTRAP-Rho-kinase-dependent neurite retraction, which is the same as Fig. 4f. The relationship was also calculated by the RD simulation (red dots).

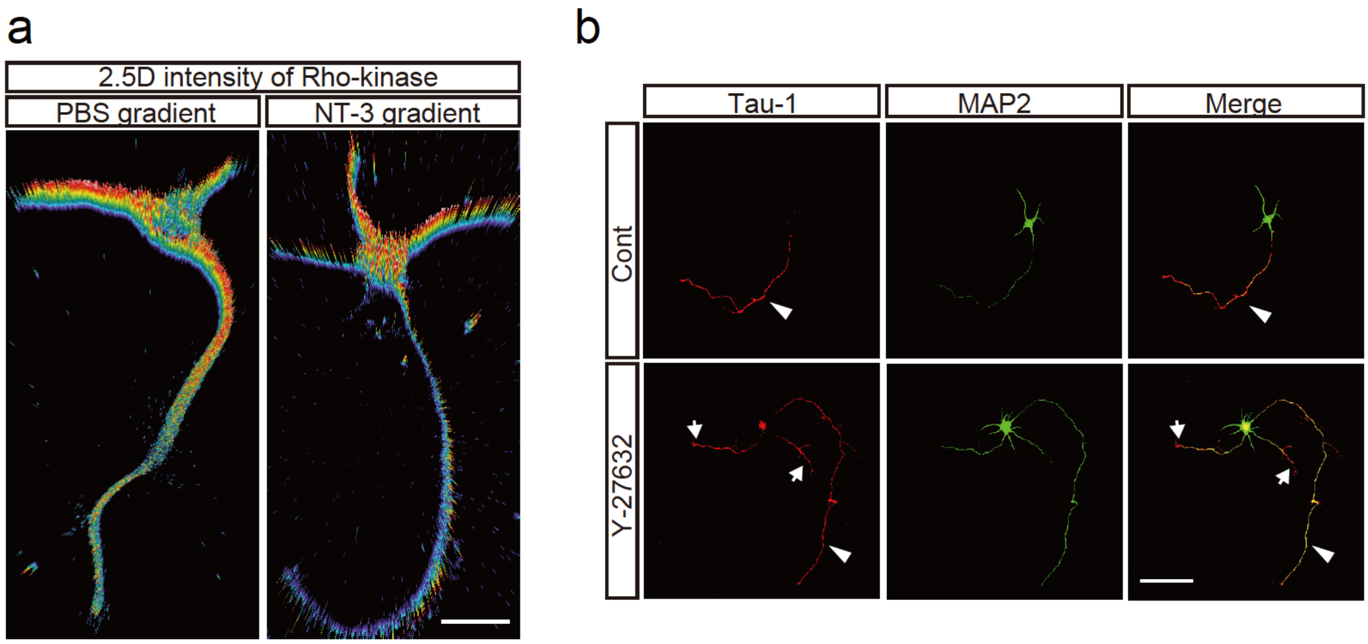

Supplementary Figure 6. Spatial distribution of Rho-kinase in hippocampal neurons and Rho-kinase regulates the maintenance of future dendrite identity (**a**) PBS (left panels) or NT-3 (right panels) was applied locally to the growth cone of the axon at 3 DIV. Neurons were immunostained with an anti-Rho-kinase antibody. The amount of Rho-kinase was analyzed by 2.5D reconstruction. Scale bars, 10 μ m. (**b**) Hippocampal neurons were treated at 3 DIV with DMSO (top) or Y-27632 (bottom) for 48 h. The neurons were co-immunostained at 5 DIV with anti-Tau-1 (red) and anti-MAP2 (green) antibodies. Multiple axons (arrows) grew out from the distal tips of the minor neurites (future dendrites) of a polarized neuron. The white arrowheads indicate the original axon.

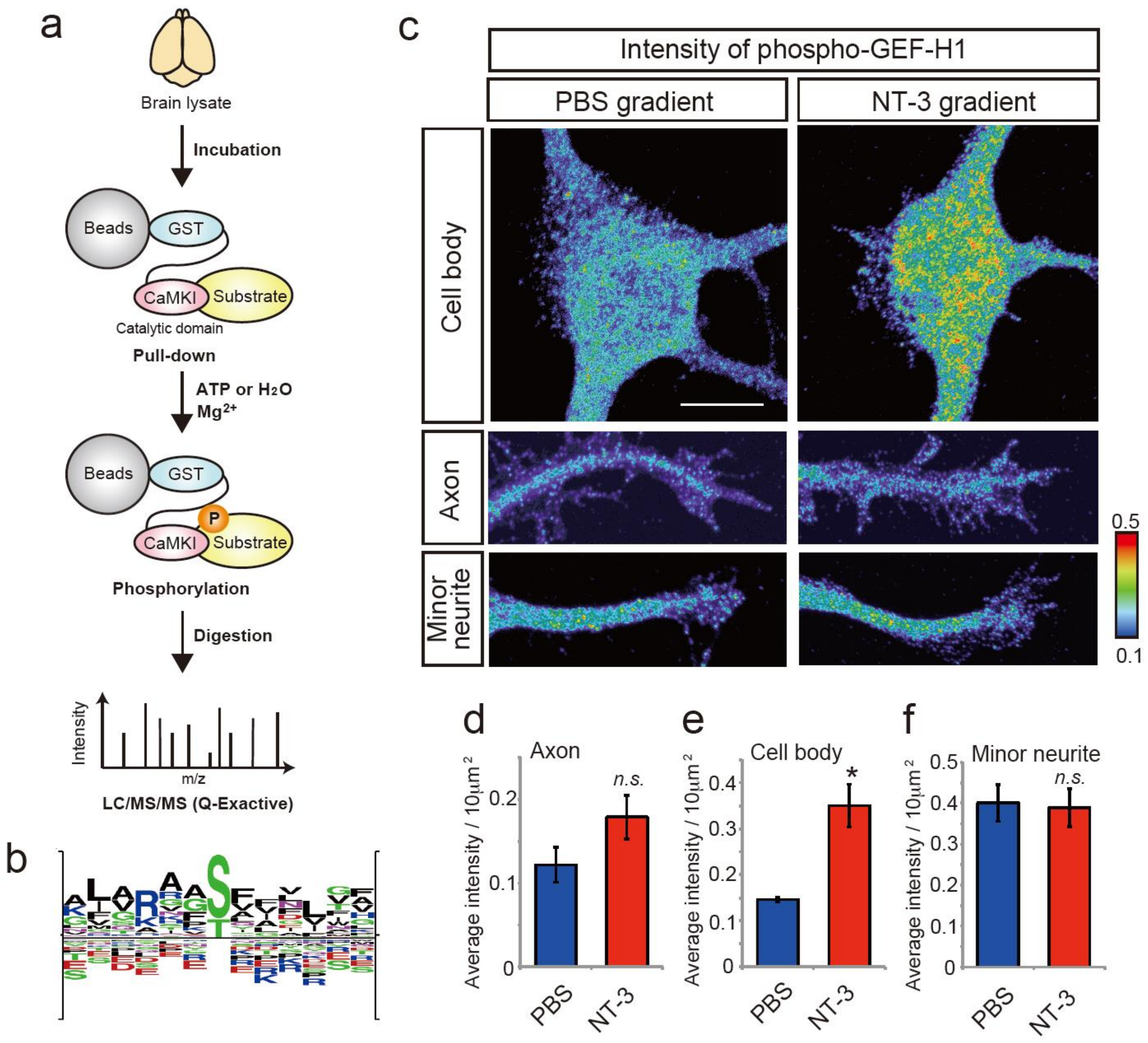

Supplementary Figure 7. GEF-H1 was identified as a novel substrate of CaMKI using the KISS method (**a**) Brain lysate was incubated with glutathione Sepharose beads coated with a GST-tagged CaMKI-cat to form a kinase-substrate complex. The bound proteins were incubated with or without ATP and Mg^{2+}. The samples were subjected to LC/MS/MS analysis. (**b**) Motif logo of candidate phosphopeptides identified from the CaMKI screening using Logo Generators (http://www.phosphosite.org). (**c**) Spatial distribution of phosphorylated GEF-H1 at Thr103 in hippocampal neurons. PBS (left panels) or NT-3 (right panels) was applied locally to the growth cone of the axon at 3 DIV. Neurons were immunostained with an anti-pT103 antibody. The pseudocolor images represent the amounts of phospho-GEF-H1 in the cell body (top), axon (middle) and minor neurite (bottom). Scale bars, 10 μm. (**d-f**) The fluorescence intensities of phospho-GEF-H1 in the axon (d), the cell body (e) and minor neurites (f) (PBS= 10, NT-3= 12 neurons from 3 independent experiments).

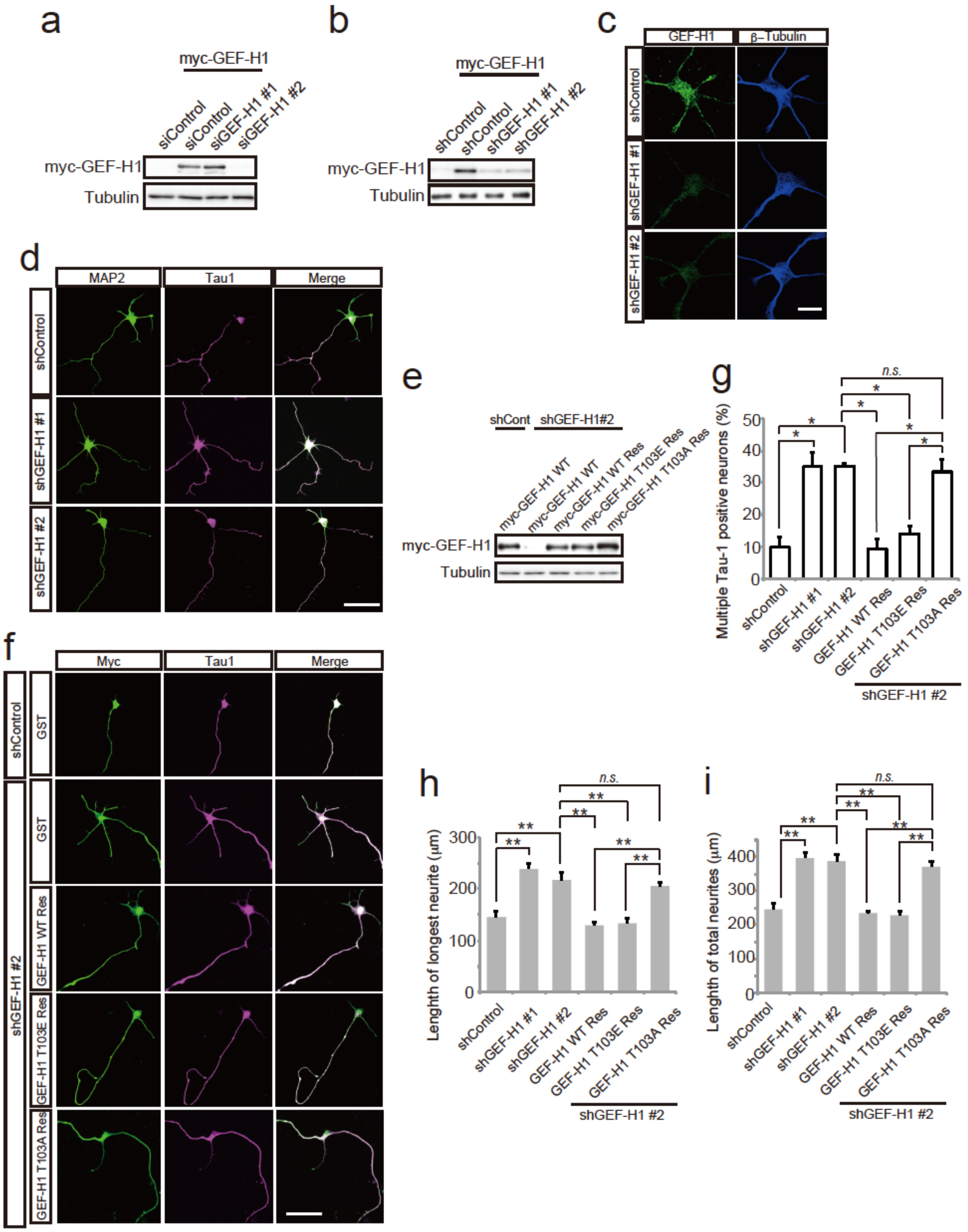

Supplementary Figure 8. The effect of GEF-H1 knockdown on neuronal polarization (**a**) Immunoblots showing knockdown of GEF-H1 with siRNA. Scramble siRNA (siControl), siRNA-GEF-H1#1 (siGEF-H1#1) or siRNA-GEF-H1#2 (siGEF-H1#2) was co-transfected with myc-GEF-H1 into Neuro2a cells. The cells were subjected to immunoblot analysis with an anti-myc antibody. Tubulin was used as a loading control. (**b**) Immunoblots revealing knockdown of GEF-H1 with shRNA. pSico-mCherry

(shControl), pSico-mCherry-shGEF-H1 #1, and -shGEF-H1 #2 were co-transfected with pEF-Cre and myc-GEF-H1 into Neuro2a cells. The cells were subjected to immunoblot analysis with an anti-myc antibody. Tubulin was used as a loading control. (**c**) Immunostaining showing knockdown of GEF-H1 in neurons. pSico-mCherry, pSico-mCherry-shGEF-H1#1 or pSico-mCherry-shGEF-H1#2 was co-transfected with Tα-Cre into hippocampal neurons at 3 DIV. The cells were immunostained with anti-GEF-H1 (left) and anti-class III β tubulin (right) antibodies. Scale bar, 20 μm. (**d**) pSico-mCherry, pSico-mCherry-shGEF-H1#1 or pSico-mCherry-shGEF-H1#2 was co-transfected with Tα-Cre into hippocampal neurons at 3 DIV. Representative images of the neurons at 4 DIV are shown. Scale bar, 50 μm. (**e**) myc-GEF-H1 WT or shRNA-resistant forms of GEF-H1 were co-transfected into Neuro2a cells with pSico-mCherry or pSico-mCherry-shGEF-H1 #2 and pEF-Cre. The cells were subjected to immunoblot analysis with an anti-myc antibody. Tubulin was used as a loading control. (**f**) Hippocampal neurons were co-transfected with pSico-mCherry, pSico-mCherry-shGEF-H1#1 or pSico-mCherry-shGEF-H1#2 and shRNA-resistant forms of GEF-H1 at 3 DIV. Representative images of the neurons at 4 DIV are shown. Scale bar, 50 μm. (**g**) Percentages of neurons with multiple Tau-1-positive axons. (**h-i**) The lengths of the longest neurite (h) and the total neurites (i) were determined (shCont= 90, shGEF-H1#1= 90, shGEF-H1#2= 90, GEF-H WT Res= 90, GEF-H1T103E Res= 90, GEF-H1T103A Res= 90 neurons from 3 independent experiments). Error bars represent the SEM. *$P < 0.05$ and **$P < 0.01$.

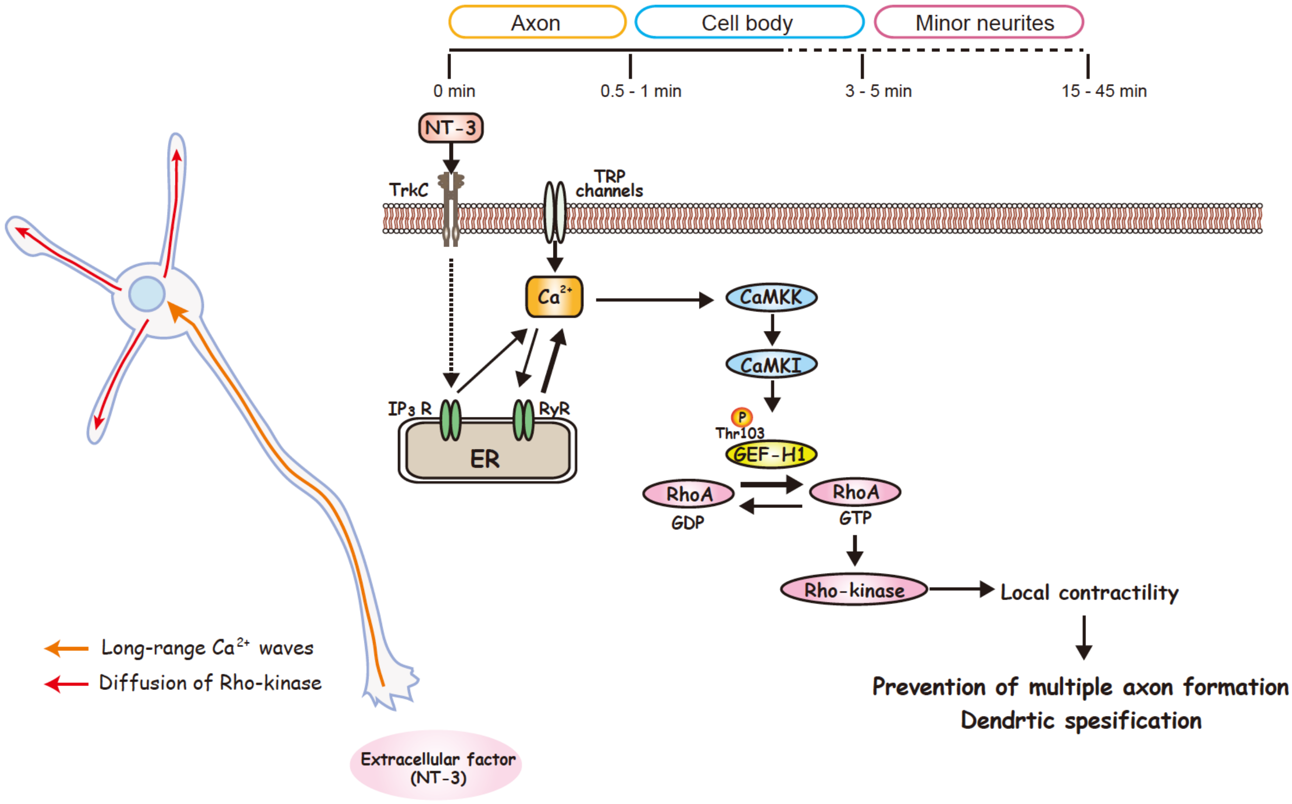

Supplementary Figure 9. Long-range inhibitory signaling regulates axon/dendrite polarity A working model of long-range inhibitory signaling for neuronal polarization. Once the axon is determined, amplification of NT-3 generates long-range Ca^{2+} waves from the axon to the cell body in a CICR-dependent manner. The long-range Ca^{2+} waves activate CaMKI, which subsequently phosphorylates GEF-H1 in the cell body. Phosphorylation of GEF-H1 induces polarized activation of RhoA/Rho-kinase, thereby activating Rho-kinase to diffuse to all minor neurites, preventing the other minor neurites from forming an axon and maintaining a future dendrite identity.

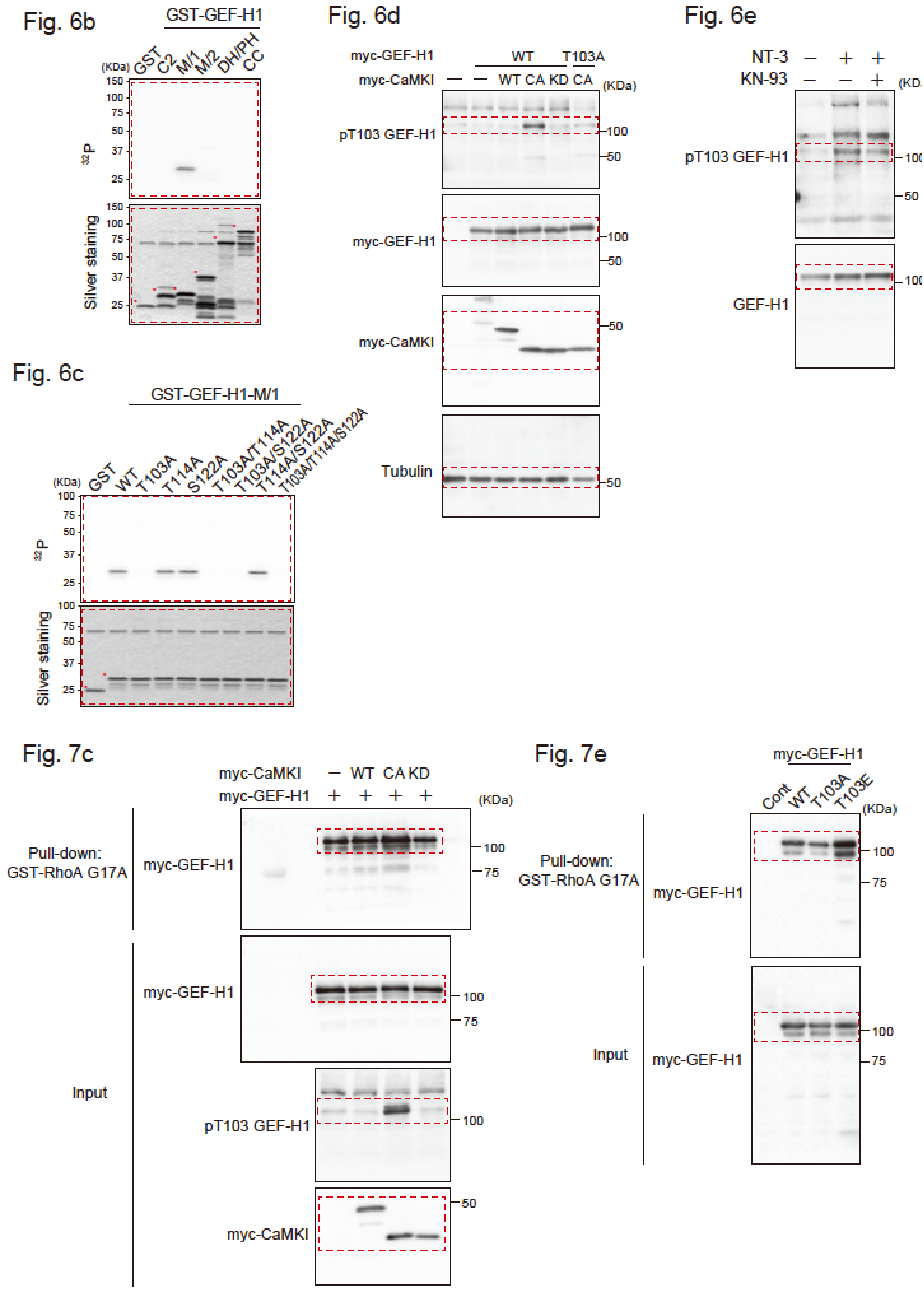

Supplementary Figure 10. Images of full-length blots

Supplementary Table 1: primers for the GEF-H1 mutants

Constructs	Primers
GEF-H1 C1	Forward 5'-ccagatcatgtctcggatcgaatccctc-3'<br>Reverse 5'-ccagatctttaggccagggtgtctttacagc -3'
GEF-H1 M/1	Forward 5'-ggagatctaactgtaccaaggtcaagcag-3'<br>Reverse 5'-ccagatctttagaagctgtcggaagggtaaat-3'
GEF-H1 M/2	Forward 5'-ggagatctcggcagtccctcctggg-3'<br>Reverse 5'-ggagatctttacaggaagctgctgtccacg-3'
GEF-H1 DH/PH	Forward 5'-ggagatccagcagcacaaaaaggaagtg-3'<br>Reverse 5'-ggagatctttaaaagtcctccctggacggg-3'
GEF-H1 CC	Forward 5'-ggagatctcctctgatcgagacagagg-3'<br>Reverse 5'-ggagatctttagctctctgaagctgtgg -3'
GEF-H1-T103A	Forward 5'-ggaacaacgctgctttgcagtctgtctccc-3'<br>Reverse 5'-gcaaagcagcgttgttcctcagcagtgcag-3'
GEF-H1-T114A	Forward 5'-gaagtaaggcgaccaccagagagcggcca-3'<br>Reverse 5'-tggtggtcgccttacttcgaagggagacag-3'
GEF-H1-S122A	Forward 5'-gccaacggctgccatttacccttccgac-3'<br>Reverse 5'-taaatggcagccgttggccgctctctggt-3'
GEF-H1-T103E	Forward 5'-ggaacaacgaggctttgcagtctgtctccc-3'<br>Reverse 5'-gcaaagcctcgttgttcctcagcagtgcag-3'
shGEF-H1 #2<br>Resistant	Forward 5'-actggaatgctagaagaattgcagat-3'<br>Reverse 5'-atctgcaattcttctagcattccagt-3'

Supplementary Tabale 2: summary of statistical analysis

Figure	Panel	Number of sample	Test used	Post-hoc test	Significance	Effect size
1e	Axon outgrowh	PBS= 14, NT-3= 15, BDNF= 16 neurons from 3 independent experiments.	One-way ANOVA	Dunnett's multiple comparison	PBS vs NT-3 or BDNF, p<0.01	Cohen's d =1.75 (NT-3), Large<br>Cohen's d =1.22 (BDNF), Large
1f	Minor neurite outgrowth	PBS= 45, NT-3= 39, BDNF= 31 neurites from 3 independent experiments.	One-way ANOVA	Dunnett's multiple comparison	PBS vs NT-3 or BDNF, p<0.05	Cohen's d =0.78 (NT-3), Medium<br>Cohen's d =0.78 (BDNF), Medium
2b	Ca2+ propagation	PBS= 10, NT-3= 16, XestosponginC= 9, Ryanodine= 15,Dantrolene= 18, SKF96365= 12 neurons from 3 independent experiments.	One-way ANOVA	Dunnett's multiple comparison	PBS vs NT-3, p<0.05	Cohen's d =1.77, Large
2c	Minor neurite outgrowth	PBS= 21, NT-3= 21, XestosponginC= 26, Ryanodine= 25, Dantrolene= 25, SKF96365= 24 neurites from 3 independent experiments.	One-way ANOVA	Dunnett's multiple comparison	PBS vs NT-3, p<0.05	Cohen's d =0.94, Large
2d	Axon outgrowh	PBS= 7, NT-3= 9, XestosponginC= 9, Ryanodine= 9, Dantrolene= 8, SKF96365= 8 neurons from 3 independent experiments.	One-way ANOVA	Dunnett's multiple comparison	PBS vs NT-3, Ryanodine, Dantrolene or SKF96365 p<0.05	Cohen's d =1.90 (NT-3), Large<br>Cohen's d =1.39 (Ryanodine), Large<br>Cohen's d =1.16 (Dantrolene), Large<br>Cohen's d =1.21 (SKF96365), Large
2f	Minor neurite outgrowth	PBS= 27, NT-3= 31, BAPTA= 47, STO-609= 45,<br>KN-93= 40 neurites from 3 independent experiments.	One-way ANOVA	Dunnett's multiple comparison	PBS vs NT-3, p<0.01	Cohen's d =0.92, Large
2g	Minor neurite outgrowth	DMSO= 42, XestosponginC= 32, Ryanodine= 37, Dantrolene= 35, SKF96365= 32 neurons from 3 independent experiments.	One-way ANOVA	Dunnett's multiple comparison	DMSO vs Xestospongin C or Ryanodine, p<0.01; DMSO vs Dantrolene or SKF96365, p<0.05	Cohen's d =0.98 (Xestospongin C), Large<br>Cohen's d =1.09 (Ryanodine), Large<br>Cohen's d =0.5 (Dantrolene), Medium<br>Cohen's d =0.56 (SKF96365), Medium
2h	Axon outgrowh	DMSO= 14, XestosponginC= 11, Ryanodine= 13, Dantrolene= 13, SKF96365= 12 neurons from 3 independent experiments.	One-way ANOVA	Dunnett's multiple comparison	DMSO vs Xestospongin C, p<0.01	Cohen's d =1.05, Large
3c	Minor neurite outgrowth	DMSO (PBS)= 16, DMSO (NT-3)= 22, C3= 23,<br>Y-27632= 28, Blebbistatin= 22 neurites from 3 independent experiments.	One-way ANOVA	Dunnett's multiple comparison	DMSO (PBS) vs DMSO (NT-3), p<0.05	Cohen's d =1.79, Large
3d	FRET efficiency	DMSO (PBS)= 37, DMSO (NT-3)= 37, BAPTA= 37, STO-609= 37, KN-93= 37 neurons from 3 independent experiments.	One-way ANOVA	Dunnett's multiple comparison	DMSO (PBS) vs DMSO (NT-3), p<0.001	Cohen's d>2.0, Large
3g	Minor neurite outgrowth<br>Photoactivation for 10 min	LOVTRAP-Cont= 40, LOVTRAP-RhoA= 23, LOVTRAP-Rho-kinase CAT= 44, LOVTRAP-Rho-kinase CAT KD= 36 neurites from 5 independent experiments.	One-way ANOVA	Dunnett's multiple comparison	LOVTRAP-Cont vs LOVTRAP-RhoA or LOVTRAP-Rho-kinase CAT, p<0.01	Cohen's d =1.01 (LOVTRAP-RhoA), Large<br>Cohen's d =0.7 (LOVTRAP-Rho-kinase CAT), Medium
	Photoactivation for 20 min				LOVTRAP-Cont vs LOVTRAP-RhoA or LOVTRAP-Rho-kinase CAT, p<0.01	Cohen's d =1.37 (LOVTRAP-RhoA), Large<br>Cohen's d =1.2 (LOVTRAP-Rho-kinase CAT), Large
	Photoactivation for 30 min				LOVTRAP-Cont vs LOVTRAP-RhoA or LOVTRAP-Rho-kinase CAT, p<0.01	Cohen's d =1.5 (LOVTRAP-RhoA), Large<br>Cohen's d =1.41 (LOVTRAP-Rho-kinase CAT), Large
	Photoactivation for40 min				LOVTRAP-Cont vs LOVTRAP-RhoA or LOVTRAP-Rho-kinase CAT, p<0.01	Cohen's d =1.89 (LOVTRAP-RhoA), Large<br>Cohen's d =1.72 (LOVTRAP-Rho-kinase CAT), Large
	Photoactivation for 50 min				LOVTRAP-Cont vs LOVTRAP-RhoA or LOVTRAP-Rho-kinase CAT, p<0.01	Cohen's d>2.0 (LOVTRAP-RhoA), Large<br>Cohen's d =1.77 (LOVTRAP-Rho-kinase CAT), Large
	Photoactivation for 60 min				LOVTRAP-Cont vs LOVTRAP-RhoA or LOVTRAP-Rho-kinase CAT, p<0.01	Cohen's d>2.0 (LOVTRAP-RhoA), Large<br>Cohen's d =1.72 (LOVTRAP-Rho-kinase CAT), Large
	After photoactivation				LOVTRAP-Cont vs LOVTRAP-RhoA,p<0.05; LOVTRAP-Cont vs LOVTRAP-Rho-kinase CAT, p<0.01	Cohen's d=1.16 (LOVTRAP-RhoA), Large<br>Cohen's d =1.05 (LOVTRAP-Rho-kinase CAT), Large
3i	Axon outgrowth<br>Photoactivation for 10 min	LOVTRAP-Cont= 8, LOVTRAP-RhoA= 9, LOVTRAP-Rho-kinase CAT=9, LOVTRAP-Rho-kinase CAT KD= 9 neurons from 3 independent experiments.	One-way ANOVA	Dunnett's multiple comparison	LOVTRAP-Cont vs LOVTRAP-Rho-kinase CAT, p<0.01	Cohen's d =1.57 (LOVTRAP-Rho-kinase CAT), Large
	Photoactivation for 20 min				LOVTRAP-Cont vs LOVTRAP-RhoA, p<0.01; LOVTRAP-Cont vs LOVTRAP-Rho-kinase CAT, p<0.05	Cohen's d>2.0 (LOVTRAP-RhoA), Large<br>Cohen's d =1.90 (LOVTRAP-Rho-kinase CAT), Large
	Photoactivation for 30 min				LOVTRAP-Cont vs LOVTRAP-RhoA or LOVTRAP-Rho-kinase CAT, p<0.05	Cohen's d>2.0 (LOVTRAP-RhoA), Large<br>Cohen's d =1.89 (LOVTRAP-Rho-kinase CAT), Large
	Photoactivation for40 min				LOVTRAP-Cont vs LOVTRAP-RhoA or LOVTRAP-Rho-kinase CAT, p<0.05	Cohen's d>2.0 (LOVTRAP-RhoA), Large<br>Cohen's d>2.0 (LOVTRAP-Rho-kinase CAT), Large
	Photoactivation for 50 min				LOVTRAP-Cont vs LOVTRAP-RhoA or LOVTRAP-Rho-kinase CAT, p<0.05	Cohen's d>2.0 (LOVTRAP-RhoA), Large<br>Cohen's d>2.0 (LOVTRAP-Rho-kinase CAT), Large
	Photoactivation for 60 min				LOVTRAP-Cont vs LOVTRAP-RhoA or LOVTRAP-Rho-kinase CAT, p<0.05	Cohen's d>2.0 (LOVTRAP-RhoA), Large<br>Cohen's d>2.0 (LOVTRAP-Rho-kinase CAT), Large
	After photoactivation				LOVTRAP-Cont vs LOVTRAP-RhoA or LOVTRAP-Rho-kinase CAT, p<0.05	Cohen's d>2.0 (LOVTRAP-RhoA), Large<br>Cohen's d>2.0 (LOVTRAP-Rho-kinase CAT), Large
5b	Minor neurite outgrowth	Cont= 17, Y−27632= 14, C3= 13, Blebbistatin= 7 neurons from 3 independent experiments.	One-way ANOVA	Dunnett's multiple comparison	Cont vs Y-27632, p<0.01; Cont vs C3 or Blebbistatin, p<0.05	Cohen's d=1.62 (Y-27632), Large<br>Cohen's d=0.95 (C3), Large<br>Cohen's d=1.38 (Blebbistatin), Large
5d	Mutiple axon formation	Cont= 91, Y−27632= 90, C3= 90, Blebbistatin= 91 neurons from 3 independent experiments.	One-way ANOVA	Dunnett's multiple comparison	Cont vs Y-27632, C3 or Blebbistatin, p<0.01	Cohen's d>2.0 (Y-27632), Large<br>Cohen's d>2.0 (C3), Large<br>Cohen's d>2.0 (Blebbistatin), Large
5e	Length of multiple axons		One-way ANOVA	Dunnett's multiple comparison	Cont vs Y-27632, C3 or Blebbistatin, p<0.05	Cohen's d=0.64 (Y-27632), Medium<br>Cohen's d=0.69 (C3), Medium<br>Cohen's d=0.74 (Blebbistatin), Medium
5f	Length of longest neurite		One-way ANOVA	Dunnett's multiple comparison	Cont vs Y-27632, C3 or Blebbistatin, p<0.01	Cohen's d>2.0 (Y-27632,) Large<br>Cohen's d>2.0 (C3), Large<br>Cohen's d>2.0 (Blebbistatin), Large
5g	Length of total neurites		One-way ANOVA	Dunnett's multiple comparison	Cont vs Y-27632, C3 or Blebbistatin, p<0.01	Cohen's d>2.0 (Y-27632), Large<br>Cohen's d>2.0 (C3), Large<br>Cohen's d>2.0 (Blebbistatin), Large
6e	pT103 GEF-H1	GEF-H1= 3, GEF-H1 + CaMKI WT= 3, GEF-H1 + CaMKI CA= 3, GEF-H1 + CaMKI KD= 3, GEF-H1 T103A + CaMKI CA = 3 indenpendet experiments	One-way ANOVA	Dunnett's multiple comparison	GEF-H1 vs GEF-H1 + CaMKI CA, p<0.05	Cohen's d>2.0, Large
6g	pT103 GEF-H1	Cont= 3, NT-3= 3,<br>NT-3 + KN-93 = 3 indenpendet experiments	One-way ANOVA	Dunnett's multiple comparison	Cont vs NT-3, p<0.05,	Cohen's d>2.0, Large
7a	Minor neurite outgrowth	siCont/PBS= 29, siCont/NT-3= 26, siGEF-H1#2/PBS= 23, siGEF-H1#2/NT-3= 26 neurites from 3 independent experiments.	One-way ANOVA	Dunnett's multiple comparison	siCont/PBS or siGEF-H1#2/NT-3 vs siCont/NT-3, p<0.01	Cohen's d=1.19 (siCont/NT-3), Large
7b	FRET efficiency	siCont/PBS= 5, siCont/NT-3= 8, siGEF-H1#2/PBS= 6, siGEF-H1#2/NT-3= 15 neurons from 3 independent experiments.	One-way ANOVA	Dunnett's multiple comparison	siCont/PBS vs siCont/NT-3, p<0.01<br>siCont/PBS vs siGEF-H1#2, P<0.01	Cohen's d=1.07 (siCont/NT-3), Large<br>Cohen's d=0.74 (siGEF-H1/NT-3), Medium
7d	GEF activity	Cont= 4, CaMKI WT= 4, CaMKI CA= 4, CaMKI KD= 4 indenpendet experiments	One-way ANOVA	Dunnett's multiple comparison	Cont vs CaMKI CA, p<0.05	Cohen's d>2.0, Large
7f	GEF activity	GEF-H1 WT= 4, GEF-H1 T103A= 4,<br>GEF-H1 T103E = 4 indenpendet experiments	One-way ANOVA	Dunnett's multiple comparison	GEF-H1 WT vs GEF-H1 T103E, p<0.01	Cohen's d>2.0, Large
7h	Mutiple axon formation	GST= 86, GEF-H1 WT= 90, GEF-H1 T103E= 88,<br>GEF-H1 T103A= 90 neurons from 3 independent experiments.	One-way ANOVA	Dunnett's multiple comparison	GEF-H1 WT vs GEF-H1 T103A, p<0.01	Cohen's d>2.0, Large
7i	Length of multiple axons		One-way ANOVA	Dunnett's multiple comparison	GST vs GEF-H1 T103E or GEF-H1 T103A, p<0.05	Cohen's d>2.0 (GEF-H1 T103E), Large<br>Cohen's d=1.18 (GEF-H1 T103A), Large
7j	Length of longest neurite		One-way ANOVA	Dunnett's multiple comparison	GST vs GEF-H1 T103E or GEF-H1 T103A, p<0.05	Cohen's d>2.0 (GEF-H1 T103E), Large<br>Cohen's d>2.0 (GEF-H1 T103A), Large
7k	Length of total neurites		One-way ANOVA	Dunnett's multiple comparison	GST vs GEF-H1 T103E or GEF-H1 T103A, p<0.05	Cohen's d>2.0 (GEF-H1 T103E), Large<br>Cohen's d>2.0 (GEF-H1 T103A), Large
8b	Distribution of cells *in vivo*	pTα-LPL-EGFP = 6, pTα-LPL-GEF-H1-WT= 9, pTα-LPL-GEF-H1-T103E = 9, pTα-LPL-GEF-H1-T103A = 4 brains from 3 independent experiments.	One-way ANOVA	Tukey's multiple comparison	Cont vs GEF-H1WT, T103E or GEF-H1 T103A, p<0.01	Cohen's d>2.0 (GEF-H1 WT), Large<br>Cohen's d>2.0 (GEF-H1 T103E), Large<br>Cohen's d>2.0 (GEF-H1 T103A), Large
8c	Morphology of cells *in vivo*		One-way ANOVA	Tukey's multiple comparison	Cont vs GEF-H1WT, T103E or GEF-H1 T103A, p<0.01	Cohen's d>2.0 (GEF-H1 WT), Large<br>Cohen's d>2.0 (GEF-H1 T103E), Large<br>Cohen's d>2.0 (GEF-H1 T103A), Large
8d	Trailling process fromation		One-way ANOVA	Tukey's multiple comparison	Cont vs GEF-H1 WT or GEF-H1 T103E, p<0.01	Cohen's d>2.0 (GEF-H1 WT), Large<br>Cohen's d>2.0 (GEF-H1 T103E), Large
8e	Leading process formation		One-way ANOVA	Tukey's multiple comparison	Cont vs GEF-H1 WT, GEF-H1 T103E, GEF-H1 T103A, p<0.01	Cohen's d>2.0 (GEF-H1 WT), Large<br>Cohen's d>2.0 (GEF-H1 T103E), Large<br>Cohen's d>2.0 (GEF-H1 T103A), Large
8g	Distribution of cells *in vivo*	pSico-mCherry= 4, pSico-mCherry-shGEF-H1 #1= 8, pSico-mCherry-shGEF-H1 #2= 4, pTα-LPL-GEF-H1-WT Res= 5, pTα-LPL-GEF-H1-T103E Res= 13,<br>pTα-LPL-GEF-H1-T103A Res = 10 brains from 3 independent experiments.	One-way ANOVA	Tukey's multiple comparison	Cont vs shGEF-H1#1, shGEF-H1#2, GEF-H1 T103E Res or GEF-H1 T103A Res,p<0.01	Cohen's d>2.0 (shGEF-H1#1), Large<br>Cohen's d>2.0 (shGEF-H1#12), Large<br>Cohen's d>2.0 (T103E Res), Large<br>Cohen's d>2.0 (T103A Res), Large
8h	Morphology of cells *in vivo*		One-way ANOVA	Tukey's multiple comparison	Cont vs shGEF-H1#1, shGEF-H1#2, GEF-H1 T103E Res or GEF-H1 T103A Res,p<0.01	Cohen's d>2.0 (shGEF-H1#1), Large<br>Cohen's d>2.0 (shGEF-H1#12), Large<br>Cohen's d>2.0 (T103E Res), Large<br>Cohen's d>2.0 (T103A Res), Large
8i	Trailling process fromation		One-way ANOVA	Tukey's multiple comparison	Cont vs GEF-H1 T103E, p<0.01	Cohen's d>2.0 (T103E Res), Large
8j	Leading process formation		One-way ANOVA	Tukey's multiple comparison	Cont vs shGEF-H1#1, shGEF-H1#2, GEF-H1 T103E Res or GEF-H1 T103A Res,p<0.01	Cohen's d>2.0 (shGEF-H1#1), Large<br>Cohen's d>2.0 (shGEF-H1#12), Large<br>Cohen's d>2.0 (T103E Res), Large<br>Cohen's d>2.0 (T103A Res), Large
S1	Axon outgrowth<br>Local application for 30 min	anti-Cont= 13, anti-NT-3= 13 neurons from 3 independent experiments.	Student's t-test		anti-Cont vs anti-NT-3, p<0.01	Cohen's d=1.54, Large
	Axon outgrowth<br>Local application for 40 min					Cohen's d=1.49, Large
	Axon outgrowth<br>Local application for 50 min					Cohen's d=1.83, Large
	Axon outgrowth<br>Local application for 60 min					Cohen's d=1.72, Large
S2b	Minor neurite outgrowth	DMSO= 67, ionomycin= 30 neurites from 3 independent experiments.	Student's t-test		DMSO vs ionomycin, p<0.05	Cohen's d=0.52 (ionomycin), Medium
S2e	Minor neurite outgrowth	DMSO=59, ionomycin=33, BAPTA= 24,<br>KN-93= 21 neurites from 3 independent experiments.	Student's t-test		DMSO vs ionomycin, BAPTA or KN-93, p<0.05	Cohen's d=1.03 (ionomycin), Large<br>Cohen's d=0.67 (BAPTA), Medium<br>Cohen's d=0.87 (KN-93), Large
S7e	pGEF-H1	PBS= 10, NT-3= 12 neurons from 3 independent experiments.	Student's t-test		PBS vs NT-3, p<0.05	Cohen's d>2.0, Large
S8g	Mutiple axon formation	shCont= 90, shGEF-H1#1= 90, shGEF-H1#2= 90,<br>GEF-H WT Res= 90, GEF-H1T103E Res= 90,<br>GEF-H1T103A Res= 90 neurons from 3 independent experiments.	One-way ANOVA	Tukey's multiple comparison	shCont vs shGEF-H1#1, shGEF-H1#2, T103A Res, p<0.05	Cohen's d>2.0 (shGEF-H1#1), Large<br>Cohen's d>2.0 (shGEF-H1#12), Large<br>Cohen's d>2.0 (T103A Res), Large
S8h	Length of longest neurite		One-way ANOVA	Tukey's multiple comparison	shCont vs shGEF-H1#1, shGEF-H1#2, T103A Res, p<0.01	Cohen's d>2.0 (shGEF-H1#1), Large<br>Cohen's d>2.0 (shGEF-H1#12), Large<br>Cohen's d>2.0 (T103A Res), Large
S8i	Length of total neurites		One-way ANOVA	Tukey's multiple comparison	shCont vs shGEF-H1#1, shGEF-H1#2, T103A Res, p<0.01	Cohen's d>2.0 (shGEF-H1#1), Large<br>Cohen's d>2.0 (shGEF-H1#12), Large<br>Cohen's d>2.0 (T103A Res), Large
